# Tigecycline pharmacodynamics in the hollow fiber system of *Mycobacterium avium*-complex lung disease and the utility of MICs and time-kill studies in drug development

**DOI:** 10.3389/fphar.2025.1682477

**Published:** 2025-11-12

**Authors:** Devyani Deshpande, Shashikant Srivastava, Tawanda Gumbo

**Affiliations:** 1 Center for Infectious Diseases Research and Experimental Therapeutics, Baylor University Medical Center, Dallas, TX, United States; 2 Division of Infectious Diseases, Department of Medicine, The University of Texas at Tyler School of Medicine, Tyler, TX, United States; 3 Department of Cellular and Molecular Biology, University of Texas Health Science Center at Tyler, Tyler, TX, United States; 4 NASOS Biotech, Dallas, TX, United States; 5 Phase Advance, Dallas, TX,, United States; 6 IMPI Group of Companies, Harare, Zimbabwe

**Keywords:** tetracycline, nontuberculous mycobacteria, pulmonary disease, PK/PD, probability of target attainment

## Abstract

**Background:**

Guideline-based therapy (GBT) drugs for *Mycobacterium avium*-complex (MAC) lung disease (LD) were chosen in part because they have low minimum inhibitory concentrations (MICs). Despite these low MICs, GBT achieves 6-month sustained sputum culture conversion in only 43% of patients.

**Methods:**

First, we co-incubated tigecycline with MAC for 7 days in time-kill studies and calculated the exposure mediating 50% of maximal effect (E_max_), or EC_50_. Next, we performed tigecycline exposure-effect studies in the hollow fiber system of MAC (HFS-MAC) inoculated with the reference ATCC#700898 isolate. Third, we performed an exposure-effect study in the HFS-MAC inoculated with five clinical isolates. Finally, the target exposure (EC_80_) was used to identify a clinical dose of inhaled tigecycline for MAC-LD in 10,000 virtual subject Monte Carlo experiments (MCE).

**Results:**

In time-kill studies, the EC_50_ was 0–24 h area under the concentration-time curve-to-MIC (AUC_0–24_/MIC) of 174 for extracellular and 4.56 for intracellular MAC (p < 0.001). In the HFS-MAC inoculated with ATCC#700898, the EC_50_ statistically differed between sampling days. However, studies with five different isolates demonstrated a stable and robust day-to-day EC_50_ (%CV = 18.18%), with an EC_80_ AUC_0–24_/MIC of 33.65. The E_max_ was 4.84 log_10_ CFU/mL. In MCE, tigecycline inhalational doses of 35–40 mg/day achieved the EC_80_ target in >90% of virtual patients, with an MIC breakpoint of 256 mg/L.

**Conclusion:**

Instead of static time-kill studies with a reference strain, inclusion of multiple MAC isolates in HFS-MAC studies improves the precision of pharmacokinetic/pharmacodynamic parameter estimates. Tigecycline administered via the inhalational route could contribute to the treatment of MAC-LD.

## 1 Introduction

Guideline-based treatment (GBT) of *Mycobacterium avium* complex (MAC) lung disease (LD), which consists of a macrolide plus rifamycin plus ethambutol, achieves 6-month sustained sputum culture conversion rates (SSCC) in only 43%–53% of patients (Kwak et al., 2017; Pasipanodya et al., 2017; Daley et al., 2020). Refractory MAC-LD is defined as failure to achieve SSCC after 6 months of GBT, which means most patients fail the GBT (Daley et al., 2020). The best predictors of poor SSCC and mortality on treatment with GBT are a high MAC bacterial burden at the start of therapy (*B*_*0*_) and the presence of cavities >2 cm in diameter (Lam et al., 2006; Kang et al., 2021; Magombedze et al., 2021). The effect of *B*_*0*_ on treatment outcomes is consistent with the inoculum effect in pharmacokinetics (PK) and pharmacodynamics (PD) science. For cavity size, drug concentrations and penetration are known to fall proportionally to the diameter, which means centers of large cavities have poor drug concentrations (Lam et al., 2006; Dheda et al., 2018; Kang et al., 2021; Magombedze et al., 2021). The intracellular hollow fiber system model of MAC-LD (HFS-MAC) can mimic the *B*_*0*_ in patient cavities, the intracellular nature of MAC in patients, and human-like antibiotic intrapulmonary PKs to mirror the SSCC reported in patients on GBT (Deshpande et al., 2010b; Schmalstieg et al., 2012; Deshpande et al., 2016; Deshpande et al., 2017a; Deshpande et al., 2017c; Ruth et al., 2019; Deshpande et al., 2020; Deshpande et al., 2023; Deshpande et al., 2024a; Deshpande et al., 2024b; Singh et al., 2024). Previously, GBT in the HFS-MAC killed between 0 and 2.3 log_10_ CFU/mL below *B*_*0*_, but the kill below *B*_*0*_ was observed in only 40% of the clinical isolates tested (Deshpande et al., 2023). Here, we used the HFS-MAC to test tigecycline for potential use in MAC-LD.

During drug development, the first screening test for candidate drug activity for MAC is the minimum inhibitory concentration (MIC). Based on the definition of resistance as an MIC ≥8 μg/mL, the MICs for MAC clinical isolates for three tetracyclines (minocycline, tigecycline, and omadacycline) led to the conclusion that 100% of *M. avium* and >87% of *M. intracellulare* isolates were resistant (Li et al., 2023). In a study by Wallace et al., 100% of 11 isolates were resistant to tigecycline and minocycline (Wallace et al., 2002). In our collection of 44 MAC isolates, resistance to tigecycline was in 90.9%, to omadacycline in 88.6%, and to minocycline in 86.4% of all isolates (Singh et al., 2025). Therefore, the general perception is that tetracyclines are “inactive” or have “no *in vitro* activity” against MAC, and thus it would be futile to test these drugs for MAC-LD (Wallace et al., 2002; Li et al., 2023). However, despite these high MICs, minocycline and omadacycline monotherapies demonstrated better microbial kill than GBT in the HFS-MAC (Ruth et al., 2019; Brown-Elliott and Wallace, 2021; 2022; Chapagain et al., 2022; Li et al., 2023). Therefore, we hypothesized that tigecycline may also show efficacy in HFS-MAC despite high MICs.

## 2 Methods

### 2.1 Materials and isolates

For MICs, static concentration exposure–effect studies were conducted in 12-well tissue culture plates. For the first HFS-MAC study, we used *M*. *avium* (American Type Culture Collection 700898, ATCC#700898). Another five clinical MAC strains (two *M avium* and three *M intracellulare*) were used in validation HFS-MAC studies. These five isolates were chosen based on their response to GBT in the HFS-MAC that mirrors the response rates encountered in patients (Deshpande et al., 2023). THP-1 monocytes (ATCC TIB-202) were used in 12-well studies. Phorbol myristate acetate (PMA), RPMI-1640 medium, and heat-inactivated fetal bovine serum (FBS) were purchased from Sigma-Aldrich (St. Louis, MO). Tigecycline was purchased from Baylor University Medical Center, Pharmacy (Dallas, TX, United States). Cellulosic HFS cartridges were purchased from FiberCell (Frederick, MD, United States).

### 2.2 MICs

We utilized the standard broth microdilution method using cation-adjusted Mueller–Hinton broth (CAMHB) supplemented with 5% oleic acid–albumin–dextrose–catalase (OADC) to determine the MICs (CLSI, 2018). The MIC assays conducted were as described by the Clinical Laboratory Standards Institute and our recent publications (CLSI, 2018; Singh et al., 2025). MICs were tested over a concentration range of 0.06 mg/L to 64 mg/L.

### 2.3 Static “time-kill” concentration-effect against extracellular and intracellular MAC

ATCC#700898 cultures in log-phase growth at a bacterial density of ∼10^5^ CFU/mL were co-incubated with tigecycline at concentrations of 0–128 mg/L in Middlebrook 7H9 broth supplemented with 10% OADC. On day 7, samples were washed, serially diluted, and inoculated onto Middlebrook 7H10 agar supplemented with 10% OADC. CFUs were recorded after incubation at 37 °C for 10 days. The experiment was performed with three replicates per concentration.

For intracellular studies, THP-1 monocytes were cultured and infected with ATCC#700898, as described previously (Deshpande et al., 2010b; Deshpande et al., 2016; Chapagain et al., 2022; Deshpande et al., 2023). Adhered and infected cells were co-incubated with tigecycline dissolved in RPMI at final concentrations of 0–8 mg/L for 7 days; the concentration range was chosen based on a pilot study. THP-1 cells were then lysed, as described before, followed by quantitation on Middlebrook 7H10 agar (Deshpande et al., 2010b; Deshpande et al., 2016; Chapagain et al., 2022; Deshpande et al., 2023). The experiment was performed with three replicates per concentration.

### 2.4 Hollow fiber system model of MAC

#### 2.4.1 Exposure-effect with ATCC#700898

HFS-MAC units were set up with a *B*_*0*_ mimicking the bacterial burden in cavitary disease by inoculating 20 mL of infected THP-1 cells in the peripheral compartment, as described in several previous publications (Deshpande et al., 2010a; Deshpande et al., 2010b; Schmalstieg et al., 2012; Deshpande et al., 2016; Deshpande et al., 2017a; Deshpande et al., 2017c; Ruth et al., 2019; Deshpande et al., 2020; Chapagain et al., 2022; Deshpande et al., 2023; Deshpande et al., 2024a; Deshpande et al., 2024b; Singh et al., 2024). We mimicked the intrapulmonary PKs of tigecycline based on a median epithelial lining fluid (ELF)-to-plasma ratio of 1.5–2.4, an ELF and alveolar macrophage half-life of 24–39 h, respectively, and an alveolar macrophage-to-ELF AUC ratio of 58.77 (Conte et al., 2005; De Pascale et al., 2014; Gotfried et al., 2017; Dimopoulos et al., 2022). Tigecycline solutions were prepared fresh each day. Seven tigecycline doses were administered once daily with target AUCs of 0–480 mg×h/L with a 2-fold decrease in doses. Non-treated HFS-MAC units served as growth controls. The target tigecycline half-life (t_1/2_) was 30 h, midway between the 24 h and 39 h reported in literature. The HFS-MAC units were repetitively sampled on days 2, 7, 14, 21, and 26 for THP-1 count and bacterial burden estimation. Bacterial burden was determined by lysing THP-1 cells, as described above. Samples were inoculated on Middlebrook 7H10 agar and in the MGIT tubes to record time-to-positivity (TTP) using the Epicenter Software. The central compartments were sampled on the last day of the study at pre-dose (0 h) and at 1 h, 6 h, 12 h, and 24 h post dosing. Tigecycline concentrations were measured using the method described previously (Ferro et al., 2016; Deshpande et al., 2019).

#### 2.4.2 Exposure-effect with five clinical MAC isolates

For drug development purposes, the US Food and Drug Administration (FDA) and European Medicines Agency (EMA) require testing at least 4–5 clinical isolates for robust PK/PD target setting (EMA, 2016). Therefore, we performed an exposure-effect HFS-MAC study with the five different clinical MAC isolates. The five isolates we used were chosen based on our interactions with regulatory authorities, who wanted a panel of five isolates that reflected the heterogeneity in response to GBT encountered in patients, to allow generalizability of findings. Each isolate had its own nontreated control (AUC/MIC = 0) and received a single daily dose of one of four different AUCs; given the different MICs, this led to a total of six AUC/MIC ratios among the five isolates (including the AUC/MIC = 0). Sampling for bacterial burden and PKs was as described in Section 2.4.1.

### 2.5 PK/PD modeling

We used the inhibitory sigmoid maximal effect (E_max_) model on each sampling day for bacterial burden (either TTP or log_10_ CFU/mL) versus drug exposures (concentration or AUC/MIC). The four parameters for this model are E_max_, the effective concentration mediating 50% of E_max_ or EC_50_, the Hill slope of H, and bacterial burden in nontreated controls (E_con_). From this, we calculated the EC_80_ as the target exposure for MCEs. Further details on the MCEs are described in the online supplementary methods.

### 2.6 Monte Carlo experiments (MCEs)

Tigecycline has been formulated for both intravenous and inhalational formulation, the latter by three separate groups in the search for less systemic toxicity (Petersen et al., 1999; Himstedt et al., 2022; Nair and Smyth, 2023). For intravenous tigecycline dosing, we used Gotfried et al. (2017). For inhaled tigecycline, we used the epithelial lining fluid (ELF) concentrations and systemic pharmacokinetic parameters generated for humans in the semi-mechanistic physiology-based pharmacokinetic model derived by Himstedt et al. (2022). Unlike Himstedt et al., we assumed that none (0%) of the drug dose would go down the gastrointestinal tract and be absorbed that way, but that all absorption would be in the respiratory tract. For inhalation therapy, the ELF compartment was specified as the central compartment, the systemic circulation was specified as peripheral compartment 1, and the remaining tissues were specified as peripheral compartment 2. The best compartmental model was then chosen based on the Akaike information criterion. The model was then used for MCE.

The inter-individual variability (as %CV) in Himstedt et al. in the inbred rats was 4.5% for clearance and 5.36% for volume, whereas in the study by Gotfried et al. in people, the variability was 17.75% for clearance and 21.26% for volume; we set these at 25% in our MCE (Gotfried et al., 2017). We examined both the intravenous route and the inhalational therapy route for doses of 0 mg, 1 mg, 10 mg, 20 mg, 30 mg, 40 mg, 50 mg, 100 mg, and 200 mg once daily. Pharmacokinetic parameters and a covariance matrix were added to the PRIOR subroutine in ADAPT 5, and the ELF and plasma concentration-time profiles were generated for each dose in 10,000 virtual subjects. The values of these estimates are shown side by side in the Results section. The AUCs so generated were examined at each MIC for probability target attainment (PTA) to achieve EC_80_ in ELF (Singh et al., 2025). Regarding toxicity, AUCs at each dose were examined for a target plasma AUC_0–24_ of >6.87 mg×h/L, demonstrated by Rubino et al. to be associated with a higher probability of nausea and vomiting (Rubino et al., 2012).

## 3 Results

### 3.1 MICs and static tigecycline concentration versus effect

The tigecycline MICs for ATCC#700898 and the five clinical isolates were as shown in Table 1. A trailing effect was observed for all isolates. One isolate had an MIC of 8 mg/L, which is considered resistant.

**TABLE 1:** Tigecycline minimum inhibitory concentrations.

Isolate	Minimum inhibitory concentration (mg/L)	Trailing effect observed over
ATCC#700898	2	3 tube dilutions
S1	1	3 tube dilutions
S2	2	3 tube dilutions
S3	1	3 tube dilutions
S4	8	3 tube dilutions
D5	4	3 tube dilutions

Table 2 shows the tigecycline inhibitory sigmoid E_max_ model parameter estimates and microbial kill below day bacterial burden (*B*_*0*_) for the extracellular and intracellular ATCC#700898 time-kill studies. Notably, the microbial kill below *B*_*0*_, H, and the EC_50_ differed significantly between intracellular versus extracellular assays, with the EC_50_ differing by a factor of 531 between the two conditions.

**TABLE 2:** Inhibitory sigmoid E_max_ parameters for intracellular versus extracellular MAC in time-kill studies.

Parameter	Extracellular	Intracellular
E_con_ log_10_ CFU/mL	10.83 ± 0.23	7.19 ± 0.12
E_max_ log_10_ CFU/mL	10.0 ± 0.37	4.33 ± 0.1
Kill below *B*_*0*_ at E_max_	1.45 ± 0.05	4.46 ± 0.17
H	1.38 ± 0.14	0.38 ± 0.17
EC_50_ mg/L	5.31 ± 0.43	0.01 ± 0.02
EC_80_ mg/L	14.5 ± 1.17	0.38 ± 0.77
r^2^	0.99	0.98

### 3.2 HFS-MAC exposure-effect results with MAC ATCC#700898

**FIGURE 1:**
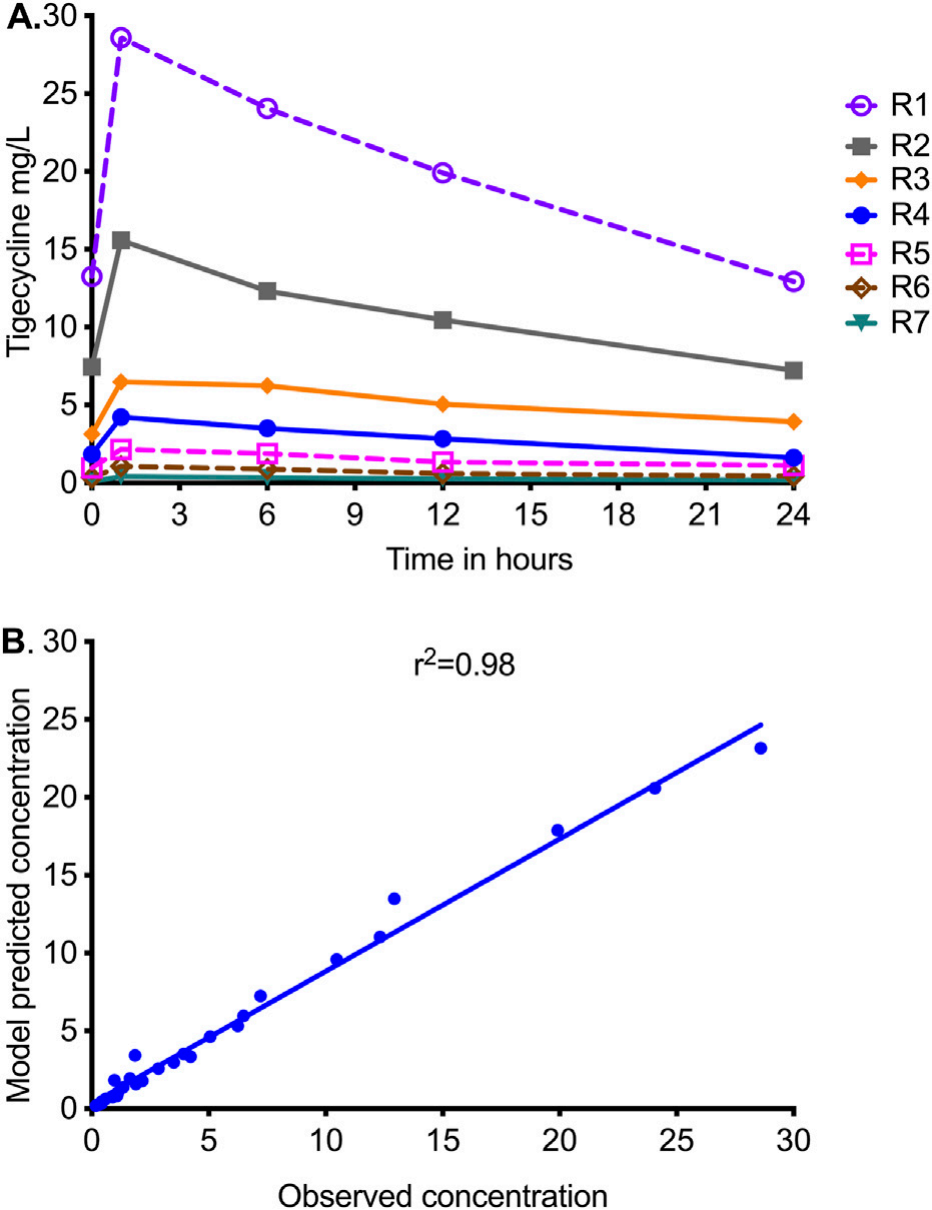
Tigecycline concentration–time profiles achieved in HFS-MAC units. R1 to R7 are descending doses of tigecycline, administered once a day. **(A)** Symbols are concentrations achieved at the different time points in each HFS-MAC. The line graphs shown are point-to-point of the observed concentrations. **(B)** The measured tigecycline concentrations versus PK model-predicted concentrations.

The tigecycline concentrations measured in the central compartment of each HFS-MAC unit are shown in Figure 1A, as are the AUC_0–24_/MIC values achieved for each regimen. PK modeling revealed an elimination rate constant (k_el_) of 0.023 ± 0.003 h^−1^, a volume of 364 ± 33 mL, and a t_1/2_ of 29.9 ± 3.7 h in the HFS-MAC. Thus, the %CV of the final PK parameters between HFS-MAC replicates was ∼10%. The observed versus model-predicted concentrations are shown in Figure 1B. The measured tigecycline concentrations versus PK model-predicted concentrations had a slope of 0.95 (95% CI: 0.92–0.99) for a one-compartment model (*r* ^2^ = 0.98), indicating minimal bias. These PK results were used to calculate the AUC_0–24_/MIC achieved for each regimen.

**FIGURE 2:**
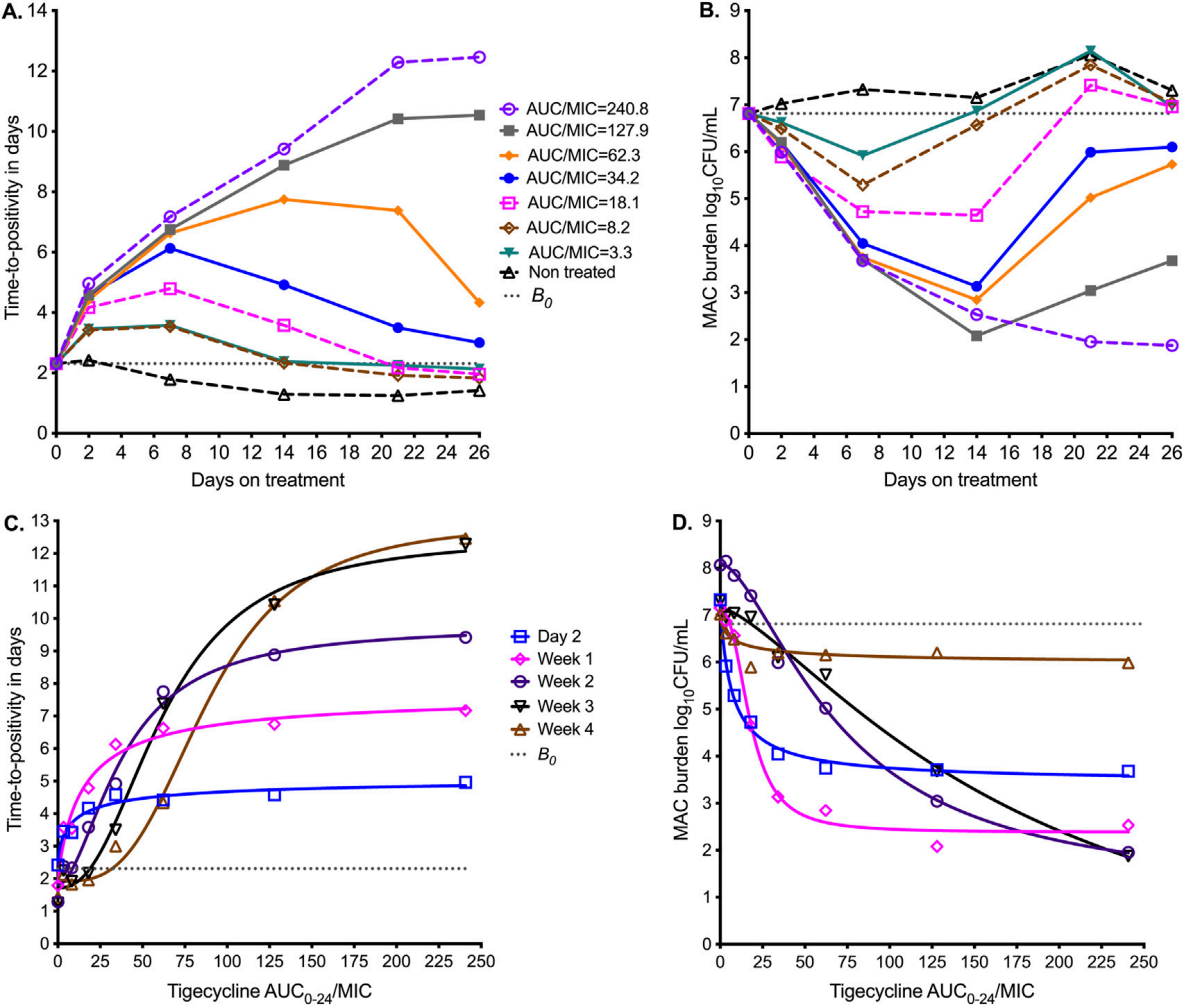
Tigecycline PK/PD at different sampling points in the HFS-MAC. Symbols are bacterial burden measurements observed at the different time points in each HFS-MAC. **(A)** Change in bacterial burden from sampling day to sampling day based on time-to-positivity readout, for each AUC/MIC exposure. The higher the time-to-positivity, the lower the bacterial burden. **(B)** The colony-forming units per mL (log_10_) readout demonstrates the same pattern. **(C)** The inhibitory sigmoid E_max_ model using time-to-positivity as a pharmacodynamic parameter. **(D)** The inhibitory sigmoid E_max_ model using log CFU/mL as a pharmacodynamic parameter readout.

Figure 2A shows the sampling day-to-day bacterial burden changes using the TTP readout from the MGIT. TTP increases as the bacterial burden decreases, and for the non-treated TTP, it decreased throughout the 26 days of study. All exposures demonstrated a biphasic effect except in the highest dose (AUC/MIC = 240.8), an initial kill demonstrated by increasing TTP, followed by rebound growth shown by decreasing TTP. The time-to-positivity readout was able to separate out the exposure response of the four highest exposures from each other at time points before day 14. Figure 2B shows results using a CFU/mL readout. The *B*_*0*_ was 6.8 log_10_ CFU/mL. All exposures, except non-treated controls or AUC/MIC = 0, killed below *B*_*0*_ and then rebounded. The highest exposure killed 4.9 log_10_ CFU/mL below *B*_*0*_. However, the CFU/mL readout was able to separate the exposure response of the four lowest exposures from each other at time points before day 14.

Inhibitory sigmoid E_max_ modeling results using the TTP readout are shown in Figure 2C, and those using CFU/mL are shown in Figure 2D. The parameter estimates for both readouts are summarized in Table 3. Table 3 shows that the EC_50_ and H changed multiple folds between sampling days and often differed by bacterial burden readout. For the TTP readout, EC_50_ changed from an AUC_0–24_/MIC of 9.49 on day 7 to 88.4 on day 26 (9.32-fold change); the H also changed in the same period. For the CFU/mL readout, EC_50_ changed from an AUC_0–24_/MIC of 7.58 on day 7 to 179.70 on day 26 (23.71-fold change). We tested the null hypothesis that EC_50_ and H were the same on all sampling days for each readout; these two parameters differed by sampling day, and therefore, we rejected the null hypothesis (*p* < 0.0001). A simple naïve pooled average of all EC_50_ for all sampling days for both TTP and CFU/mL readouts was an AUC_0–24_/MIC of 49.90 ± 54.4, which translates to a mean EC_80_ AUC_0–24_/MIC of 126.4 (95%CI: 30.45–222.3), indicating that this is an imprecise estimate.

**TABLE 3:** Pharmacokinetic/pharmacodynamic parameter estimates and standard error using two readouts.

Parameter	Day 2	Day 7	Day 14	Day 21	Day 26
TTP READOUT					
E_con_ time-to-positivity in days	2.43 ± 0.24	1.90 ± 0.42	1.81 ± 0.33	1.72 ± 0.27	1.92 ± 0.21
E_max_ time-to-positivity in days	2.70 ± 0.67	5.77 ± 0.97	7.95 ± 0.77	10.78 ± 0.81	11.02 ± 0.71
H	0.67 ± 0.35	0.89 ± 0.30	1.80 ± 0.43	2.37 ± 0.47	3.25 ± 0.54
EC_50_ AUC_0–24_/MIC	9.49 ± 7.59	15.10 ± 6.58	38.77 ± 5.61	62.96 ± 6.16	88.40 ± 6.61
r^2^	0.95	0.97	0.99	>0.99	>0.99
Corrected Akaike information criterion score	11.46	20.88	21.94	22.51	21.16
CFU/ML READOUT					
E_con_ log_10_ CFU/mL	7.03 ± 0.08	7.31 ± 0.14	7.08 ± 0.18	8.1 ± 0.05	7.16 ± 0.15
E_max_ log_10_ CFU/mL	1.08 ± 0.22	3.87 ± 0.24	4.70 ± 0.27	7.04 ± 0.23	8.84 ± 3.32
H	0.65 ± 0.32	0.92 ± 0.15	2.45 ± 0.49	1.61 ± 0.10	1.39 ± 0.36
EC_50_ AUC_0–24_/MIC	7.58 ± 5.45	6.93 ± 1.16	17.85 ± 1.60	72.2 ± 3.35	179.70 ± 98.63
r^2^	0.98	>0.99	>0.99	>0.99	>0.99
Corrected Akaike information criterion score	27.89	3.111	11.83	26.43	10.34

### 3.3 HFS-MAC exposure-effect results with five clinical strains

**FIGURE 3:**
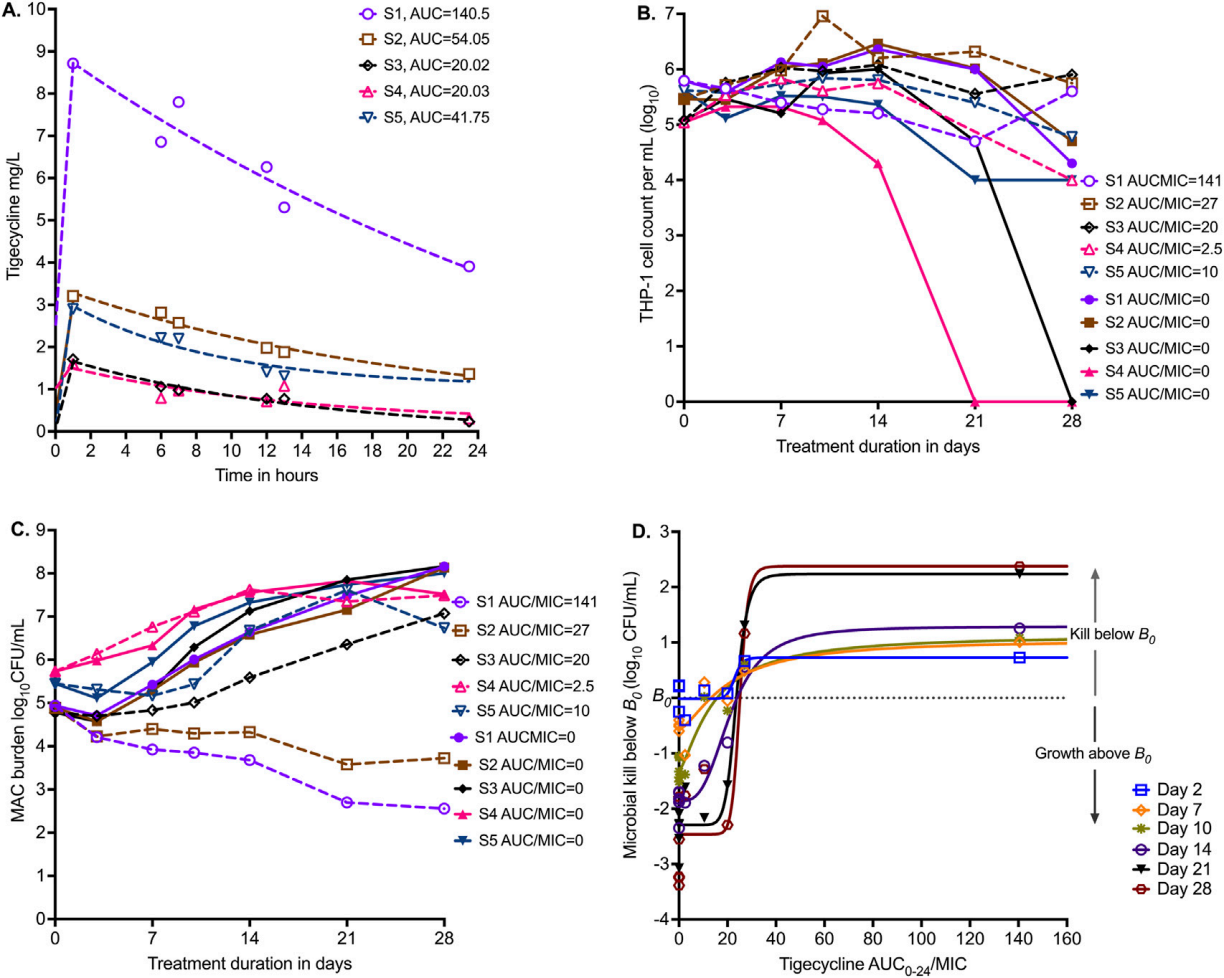
Tigecycline PK/PD in the HFS-MAC with five MAC strains. **(A)** Tigecycline 24 h concentration–time profiles based on measurement in each HFS-MAC unit. The line graphs are pharmacokinetic model-based output. Non-treated controls all had 0 concentration and are not shown. **(B)** Infected THP-1 cells are killed by MAC, based on the virulence of the isolate. Non-treated systems, that is, AUC/MIC = 0, are shown as solid symbols. **(C)** Infectivity is reflected by the variability in *B*_*0*_. The effect of each of five different exposures (one for each isolate) versus no drug treatment on day-to-day changes in bacterial burden is shown. **(D)** No single isolate was treated with a full dose–response range; they all received a single daily AUC/MIC exposure (in addition to each having a non-treated control). Shown is the inhibitory sigmoid E_max_ model that used data from all five isolates pooled for each sampling day (i.e., co-modeled), with a pharmacodynamic readout of kill below *B*_*0*_.

Figure 3A shows the results of PK modeling for the HFS-MAC units with five clinical strains,. The MICs of each isolate are shown in Table 1. Figure 3B shows the viability of THP-1 cells during the experiment, with the nontreated HFS-MAC illustrating the heterogeneity of the different MAC isolates in killing the THP-1 cells. Figure 3C shows that the *B*_*0*_ ranged from 4.8 log_10_ CFU/mL to 5.73 log_10_ CFU/mL. Thus, despite the same multiplicity of infection, the *B*_*0*_ differed between the isolates due to variations in infectivity. The growth patterns in nontreated controls also differed by isolate. Figure 3C shows changes in bacterial burden for each clinical strain versus the tigecycline AUC_0–24_/MIC achieved in the respective HFS-MAC units.

We co-modeled all five isolates (each one receiving a different AUC/MIC exposure) in one inhibitory sigmoid E_max_ equation. Given the differences in *B*_*0*_ and E_con_ (bacterial burden in non-treated controls), we used microbial kill below *B*_*0*_ for each isolate for each sampling day, with results shown in Figure 3D and Table 4. Table 4 demonstrates virtually no change in EC_50_ from day-to-day sampling with high precision (18.18 %CV). We tested the null hypothesis that EC_50_ and H were the same on all sampling days for each readout. These two parameters did not differ by sampling day, and therefore, we did not reject the null hypothesis (*p* = 0.985). The average EC_80_ across sampling days was an AUC_0–24_/MIC of 33.65 (95% CI: 23.73–43.96), which is a much narrower confidence interval than in the ATCC#700898-inoculated HFS-MAC experiment.

**TABLE 4:** Inhibitory sigmoid E_max_ estimates with kill below B_0_ as the pharmacodynamic parameter.

Sampling Day	E_con_ log_10_ CFU/mL		E_max_ log_10_ CFU/mL		H		EC_50_ AUC_0–24_/MIC		*r* ^2^	AICc
	Estimate	SEM	Estimate	SEM	Estimate	SEM	Estimate	SEM		
Day 2	−0.02	0.12	0.74	0.22	14.18	20.10	22.91	4.95	0.75	0.48
Day 7	−0.57	0.12	1.62	0.33	1.50	1.18	19.89	8.50	0.87	−10.31
Day 10	−1.31	0.13	2.45	0.31	1.39	0.65	14.24	4.22	0.94	−10.00
Day 14	−1.86	0.12	3.15	0.25	3.12	1.21	21.29	2.26	0.96	−9.60
Day 21	−2.29	0.16	4.53	0.33	10.08	3.10	23.63	1.16	0.97	−2.49
Day 28	−2.46	0.30	4.84	0.64	14.70	17.39	25.09	2.57	0.91	11.91

SEM−standard error of the mean.

### 3.4 Monte Carlo experiments (MCE) of intravenous and inhalation tigecycline doses

**FIGURE 4:**
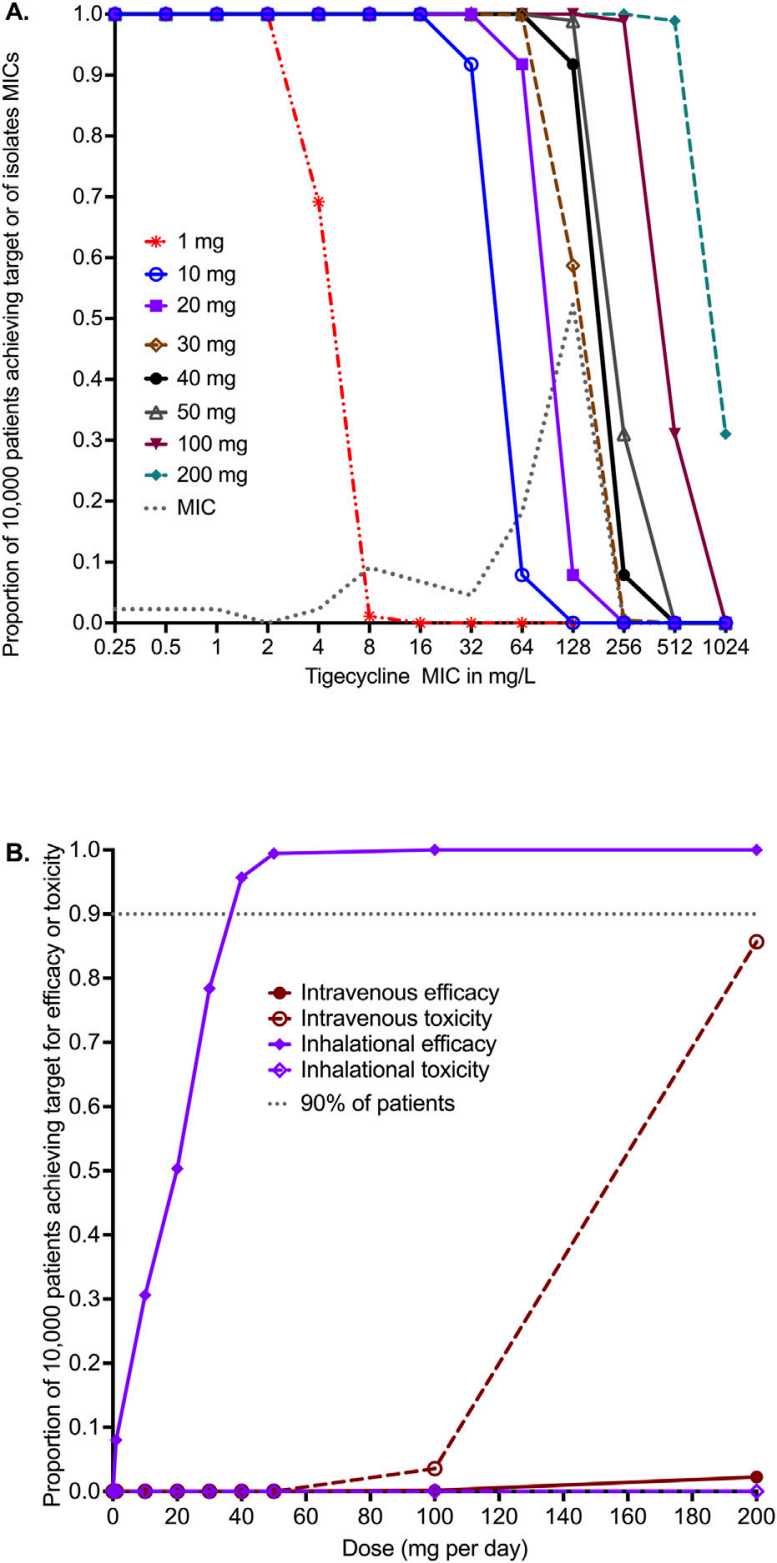
*in silico* dose finding for intravenous and inhaled tigecycline. **(A)** Probability of target attainment (PTA) for inhaled tigecycline doses. **(B)** The cumulative fraction of response (CFR) for intravenous dosing versus inhaled dosing for both efficacy and toxicity thresholds.

The PK parameter estimates and variance in the domain of input are compared to MCE-generated values in virtual subjects in Table 5 and demonstrate good recapitulation of parameters reported in patients. We examined tigecycline doses of 1 mg, 10 mg, 20 mg, 30 mg, 40 mg, 50 mg, 100 mg, and 200 mg once daily for the ability to achieve or exceed EC_80_ in ELF. The probability of target attainment (PTA) for the intravenous doses up to 1 mg, 10 mg, 20 mg, 30 mg, 40 mg, 50 mg, 100 mg, and 200 mg was zero within the tigecycline MIC range examined, which was based on the MIC distribution from our work elsewhere (Singh et al., 2025). The PTAs for inhaled doses were as shown in Figure 4A. The inhalation doses of 1 mg and 10 mg showed that PTA falls below 90% before the modal MIC of 128 mg/L, while the dose of 40 mg achieved a PTA of >90% at that MIC. The clinical susceptibility breakpoint for the dose of 30 mg/day is an MIC of 128 mg/L, and the breakpoint for the 40 mg/day and 50 mg/day doses was an MIC of 256 mg/L.

**TABLE 5:** Monte Carlo simulation experimental pharmacokinetic model output versus domain of input values**.**

PK Parameter	Parameters in the domain of input		Output in 10,000-subject simulation	
	Parameter estimate	IIV (%)	Parameter estimate	IIV (%)
ELF clearance in L/h	0.68 × 10^−2^	25.0	0.68 × 10^−2^	25.24
ELF volume in L	0.07 × 10^−2^	25.0	0.07 × 10^−2^	24.98
K_a_ (from lung to systemic)	1.002	25.0	0.99	39.45
Systemic clearance L/h	23.1	25.0	22.97	25.10
Systemic volume in L	315	25.0	314.6	25.31
Lung AUC_0–24_ for inhaled 10 mg dose (mg×h/L)			1,520	25.19
Lung AUC_0–24_ for inhaled 30 mg dose (mg×h/L)			4,559	25.19
Lung AUC_0–24_ for inhaled 40 mg dose (mg×h/L)			6,079	25.19

IIV = inter-individual variability.

The cumulative fraction of responses (CFR) for the lung AUC_0–24_/MIC≥33.55 is shown in Figure 4B for both intravenous and inhalational doses. The intravenous dose of 200 mg/day achieved the EC_80_ target in ELF in only 2.26% of 10,000 virtual patients, lower than the 1 mg/day inhalational doses, which achieved the CFR in 8.49% of patients. Figure 4B shows that 90% of patients would achieve the target at a dose of 35 mg/day, and 95.70% would achieve the target at a dose of 40 mg/day. No inhalational dose tested in the MCE was predicted to achieve the plasma AUC_0–24_ of >6.87 mg×h/L associated with a higher probability of toxicity. The intravenous dose of 100 mg/day was predicted to achieve the AUC associated with a higher risk of adverse effects in 3.57% of patients, whereas the toxicity probability with the 200 mg/day dose increased to 85.67% in the 10,000 virtual patients.

## 4 Discussion

Like other bacteria, the first step in the MAC drug development program is testing for MICs. Based on that, >90% of MAC clinical isolates were considered resistant to tigecycline, suggesting that the drug has poor activity and should not be used for MAC-LD (Wallace et al., 2002; Li et al., 2023; Singh et al., 2025). Such susceptibility studies were also used to make decisions for the use of minocycline and omadacycline, to which the isolates were considered resistant. However, despite these high MICs, in an open-label clinical trial of minocycline combined with clarithromycin and clofazimine, the SSCC rate was 64%, which is better than the GBT (Roussel and Igual, 1998). Moreover, both minocycline and omadacycline monotherapies demonstrated better microbial kill than GBT in the HFS-MAC (Ruth et al., 2019; Brown-Elliott and Wallace, 2021; 2022; Chapagain et al., 2022; Li et al., 2023). The discrepancy between high MICs and good efficacy against MAC is likely due, in part, to two “technical” reasons. First, the chemical instability or degradation rate of tetracyclines in the growth medium is faster than the MAC doubling time (Chapagain et al., 2022; Singh et al., 2025). Second, the discrepancy could be because MICs are performed using extracellular assays, while in patient lungs, MAC is predominantly intracellular (Ushiki et al., 2011; Hibiya et al., 2012). Minocycline, tigecycline, and omadacycline achieve alveolar cell-to-serum AUC penetration ratios of 25–77-fold (Conte et al., 2005; Gotfried et al., 2017). In our static concentration experiments, the tigecycline EC_50_ for intracellular MAC was 531-fold lower than for extracellular MAC, suggesting that extracellular assays may not be as predictive as regards to potency against MAC. While MICs must continue to be used in the drug development process, we propose that at least PK/PD studies must be performed first before a candidate drug is considered ineffective.

All MIC readouts were associated with the trailing effect. The trailing effect is the reduced but persistent growth of bacteria through serial microdilutions, encountered in broth-based MICs, and is commonly encountered in tetracyclines and mycobacteria (Shankar et al., 2022; Singh et al., 2025; Terschlüsen et al., 2025). The reasons are unknown but likely include the rapid degradation of tetracyclines in the MIC assay broth, or, as is the case in yeasts, upregulation of some genes involved in drug resistance at some concentrations (Lee et al., 2004). Regardless of the mechanism, this phenomenon leads to imprecision in reading MICs and interpreting the results as regards to susceptibility or resistance. It could also contribute to the discrepancy between tetracycline MICs and observed effects.

As part of a more complete PK/PD data package, guidance by regulatory authorities includes co-incubation of bacteria with static concentrations of the candidate drug, including time-kill curves (EMA, 2016). For MAC, we have proposed the use of intracellular and extracellular “time-kill” assays as a more “PK/PD” approach than MIC (Deshpande et al., 2017b). From a PK/PD standpoint, the question is whether parameter values such as EC_50_ or EC_80_ identified in “time-kill” assays contribute to those identified in the HFS-MAC (or in mice, for that matter) or in patients. As an example, in Table 2, if the concentrations are converted to AUC_0–24_/MIC, the EC_80_ for extracellular becomes 174, and that for intracellular becomes 4.56. Both figures differ from the EC_80_ of AUC_0–24_/MIC of 50.12 on day 7 in the HFS-MAC. This is important for two reasons. First, the only directly transferable information between the preclinical models and patients is the PK/PD targets (EC_50_ and EC_80_). Second, the whole point of PK/PD studies is the identification of target exposure and dosing schedule for clinical application. It is not possible to perform dose-fractionation using these static concentration assays. Therefore, we question the PK/PD utility of performing these static concentration assays in the drug development process for MAC.

Here, as elsewhere, we found that the EC_80_ target values with ATCC#700898 isolate “wobble” and vary across a wide range between sampling days (Musuka et al., 2013; Chapagain et al., 2022; Deshpande et al., 2023). This wobble makes it difficult to identify the target exposure value to use in dose selection. Our results here and elsewhere, and from others, suggest that the ATCC#700898 isolate likely provides a misestimate of PK/PD target exposure. Similarly, in the HFS-MAC inoculated with ATCC#700898, the GBT combination killed 2.1 log_10_ CFU/mL below *B*_*0*_, but the effect on five clinical isolates was such that GBT killed below *B*_*0*_ in only 2/5 strains and failed in the remaining three, very similar to what is encountered in patients (Kwak et al., 2017; Pasipanodya et al., 2017; Daley et al., 2020; Deshpande et al., 2023). Here, tigecycline killed 4.9 log_10_ CFU/mL ATCC#700898 below *B*_*0*_, but had lower effect in the five clinical isolates in the HFS-MAC. This means that, similar to rapidly growing bacteria and both the US FDA and EMA Guidance, HFS-MAC studies should include more than four isolates for a more precise estimation of PK/PD target exposures (EMA, 2016).

Finally, using the EC_80_ target from the five clinical isolates in the HFS-MAC study, we identified intravenous and inhalational tigecycline doses for the treatment of MAC-LD. The EC_80_ target was chosen based on work in tuberculosis whereby comprehensive analyses [1] of preclinical models and patients demonstrated that the EC_80_ was mathematically invariant, and [2] EC_80_-based target exposures, optimal doses, and susceptibility breakpoints were identical to those identified by agnostic artificial intelligence algorithms (which did not use inhibitory sigmoid E_max_) in 20 clinical studies (Liu et al., 2023). The intravenous tigecycline doses performed poorly. However, an inhaled dose of 35–40 mg/day was able to achieve the exposure target in >90% of the 10,000 virtual patients, with a PK/PD MIC breakpoint of 256 mg/L. This is where the MIC distribution should be used: to identify optimal dose and PK/PD-based breakpoints. At the 35 mg inhaled dose, this MIC breakpoint was greater than the MAC MIC_90_, which means > 90% of isolates were susceptible (Li et al., 2023). This is also a relatively low dose compared to the intravenous route, and, given the poor absorption from the respiratory tract, systemic concentrations will be low. This leads to a caveat, which is that we assumed none (0%) of the drug dose would go down the gastrointestinal tract and be absorbed that way, given the efficiency of nebulizers and metered dose inhalers. It is possible that some of the drug may go down the gastrointestinal tract; however, oral absorption is poor. On the other hand, inhalation itself may have adverse events, including dysphonia and bronchoconstriction. Thus, clinical studies will need to be performed to determine the safety of this dose.

Recently, the US Food and Drug Administration (FDA) and the European Medicines Agency (EMA) issued a new roadmap stating that “we need better tools to assess safety, efficacy, and pharmacology of drugs and therapeutics without traditional animal models” and that such tools must be of “high relevance to human biology” (EMA, 2025; FDA, 2025). Of relevance to MAC therapeutic outcomes are the following factors of relevance to human biology: intracellular infection, *B*_*0*_, cavity size, and human-like antibiotic intrapulmonary PKs. First, we achieved the typical *B*_*0*_ encountered in patient cavities of 4.23–6.2 log_10_ CFU/mL (Ushiki et al., 2011). The size of the HFS-MAC cartridge holds cultures equivalent to a cavity of 2 cm. It is important to recapitulate the human intrapulmonary PKs: as an example, the animal half-life of 2 h for levofloxacin in the HFS model of anthrax in mice and macaques lung disease led to therapy failure and resistance emergence, whereas at the human half-life of 7.5 h, there was persistent reduction in bacterial burden (Deziel et al., 2005; Kao et al., 2006). Here, we also show that it will be important to test MAC isolates that have different infectivity and virulence to allow for the generalizability of results. Moreover, these isolates also reflected the same heterogeneity of response rates as seen in patients on GBT. Despite the heterogeneity, the collective isolates gave a more robust PK/PD target exposure calculation than the ATCC reference isolate alone. The current study informs how to optimize such “new approach methodology” (FDA, 2025) for MAC lung disease.

There are several limitations in our studies. First, given the degradation of tigecycline in agar, we could not characterize the PK/PD parameters associated with resistance suppression. Second, we did not employ a complete exposure–response surface for each of the five clinical isolates. However, we borrowed the technique used in murine studies by Craig and Andes for rapidly growing Gram-negative bacilli and Gram-positive cocci (Craig and Andes, 2008). They first identified a full exposure response using the standard laboratory strain, used a limited number of doses in a larger number of clinical isolates with different MICs, and then co-modeled all results in a single inhibitory sigmoid E_max_ model. Third, in our MCE, we used ELF PKs based on a physiologically based pharmacokinetic (PBPK) model, and not from direct sampling of ELF in patients after administration of inhalational doses. However, even when we used the clearance rates based on rat ELF PKs from direct sampling or observations (usually faster clearance than achieved in people), the PTAs did not change significantly.

## 5 Conclusion

MIC distributions, which showed >90% of MAC isolates would be resistant to tigecycline, and static concentration versus effect studies appear not to be as informative as HFS-MAC with regards to tigecycline microbial kill below *B*_*0*_ and PK/PD target exposure identification. Inhaled tigecycline at 35–40 mg/day was predicted to achieve target exposures in lungs in MCEs and should be tested as a therapy for MAC-LD, after further preclinical studies. The PK/PD susceptibility breakpoint MIC for inhaled tigecycline dosing was determined as 256 mg/L. Finally, for precise PK/PD target exposure determination, multiple MAC strains should be tested in HFS-MAC.

## Data Availability

The raw data supporting the conclusions of this article will be made available by the authors, without undue reservation.
